# Acoustical cues for perception of emotional vocalizations in rats

**DOI:** 10.1038/s41598-019-46907-0

**Published:** 2019-07-22

**Authors:** Yumi Saito, Ryosuke O. Tachibana, Kazuo Okanoya

**Affiliations:** Department of Life Sciences, Graduate School of Arts and Sciences, The University of Tokyo, 3-8-1 Komaba, Meguro-ku, Tokyo Japan

**Keywords:** Auditory system, Psychology, Animal behaviour

## Abstract

The ultrasonic vocalizations of rats can transmit affective states to listeners. For example, rats typically produce shorter calls in a higher frequency range in social situations (pleasant call: PC), whereas they emit longer calls with lower frequency in distress situations (distress call: DC). Knowing what acoustical features contribute to auditory discrimination between these two calls will help to better characterize auditory perception of vocalized sounds in rats. In turn, this could lead to better estimation of models for processing vocalizations in sensory systems in general. Here, using an operant discrimination procedure, we examined the impact of various acoustical features on discriminating emotional ultrasonic vocalizations. We did this by systematically swapping three features (frequency range, time duration, and residual frequency-modulation pattern) between two emotional calls. After rats were trained to discriminate between PC and DC, we presented probe stimuli that were synthesized calls with one or two acoustical features swapped, and examined if the rats judged these calls as either PC or DC. The results revealed that all features were important for discrimination between the two call types, but frequency range provided the most information for discrimination. This supports the hypothesis that while rats utilize all acoustical features to perceive emotional vocalizations, they considerably rely on frequency cues.

## Introduction

Ultrasonic vocalizations (USV) in adult rats are signals that convey social and emotional information. These vocalizations fall into two predominant categories, characterized by the frequency range of the spectral peak (either around 22 or 50 kHz). The higher frequency sounds (~50 kHz) are produced when the animal is in pleasant situations (“pleasant call”; PC), for example, when socially approaching a conspecific^1,2^, engaging in rough-and-tumble play^3^, or being tickled^4^. On the other hand, rats emit the lower frequency (~22 kHz) vocalization when they are in distress situations (“distress call”; DC), such as when there is a risk of predation^5^ or aversive stimuli present (electric shock;^6^ startling noises;^7^ air puffs;^8^ human handling^9^). In addition, males that are socially defeated after a same-sex confrontation also emit these calls with a defensive posture and freezing behavior^10^.

These two calls not only reflect the caller’s situation but also transmit affective and social information to other conspecifics^3^. Playback of PC facilitates approaching social information to other conspecific^11^. On the other hand, playback of DC promotes fear-induced activities (escape or freezing) as if animals had encountered an aversive situation^12^. Moreover, DC emitted as a submissive signal by a defeated male reduces aggressive behavior from the dominant rat^13^. In addition, if rats learn to discriminate different cues in order to receive a reward or avoid a punishment, they tend to judge an ‘ambiguous cue’ (e.g. the presentation of the intermediate stimulus between the reward and punishment cue: cognitive bias task) based on what type of call is played back. They tend to treat an ambiguous cue as a reward/punishment one when PC/DC is played back (optimistic/pessimistic cognitive bias)^14^.

Three distinctive acoustical features account for the differences between PC and DC: frequency range, duration, and frequency modulation (FM) patterns. The PC has a frequency range of 35−70 kHz^15^, a short duration of 3–65 ms^13^, and shows various modulation patterns (bandwidth 3−50 kHz^16^). On the other hand, DC has a frequency range of 18−32 kHz^17^, a long duration of 300–3400 ms^13^, and shows a flat pattern with little frequency change (bandwidth 1–5 kHz^17^). Between PC and DC, the difference is shown in FM patterns, the temporal change of patterns in a single call (a unit of sound separated by silence on either side). Examples of FM patterns include trills (rapid frequency oscillations), upward/downward ramps (monotonically increasing/decreasing in frequency, with a mean positive/negative slope), flat patterns (near-constant frequency calls)^16^.

Since rats show different responses when hearing PC or DC as described above, they should be able to discriminate the acoustical differences between USV sounds. To understand how and to what extent they can discriminate their own vocal signals will inform how acoustical features can drive animal cognition and behavior. In addition, by investigating the relationship between physical characteristics of vocal signals and animal responses, we can make predictions about the mechanism of neural processing (e.g., how rat auditory systems code their vocalizations). Thus, we assessed auditory discrimination of PC and DC with operant conditioning to determine the relative contribution of different acoustical features: frequency range, duration, and FM pattern. Using probe stimuli, which partially simulate the two emotional calls, we attempted to detect the salient acoustic features for discrimination.

## Results

**Figure 1:**
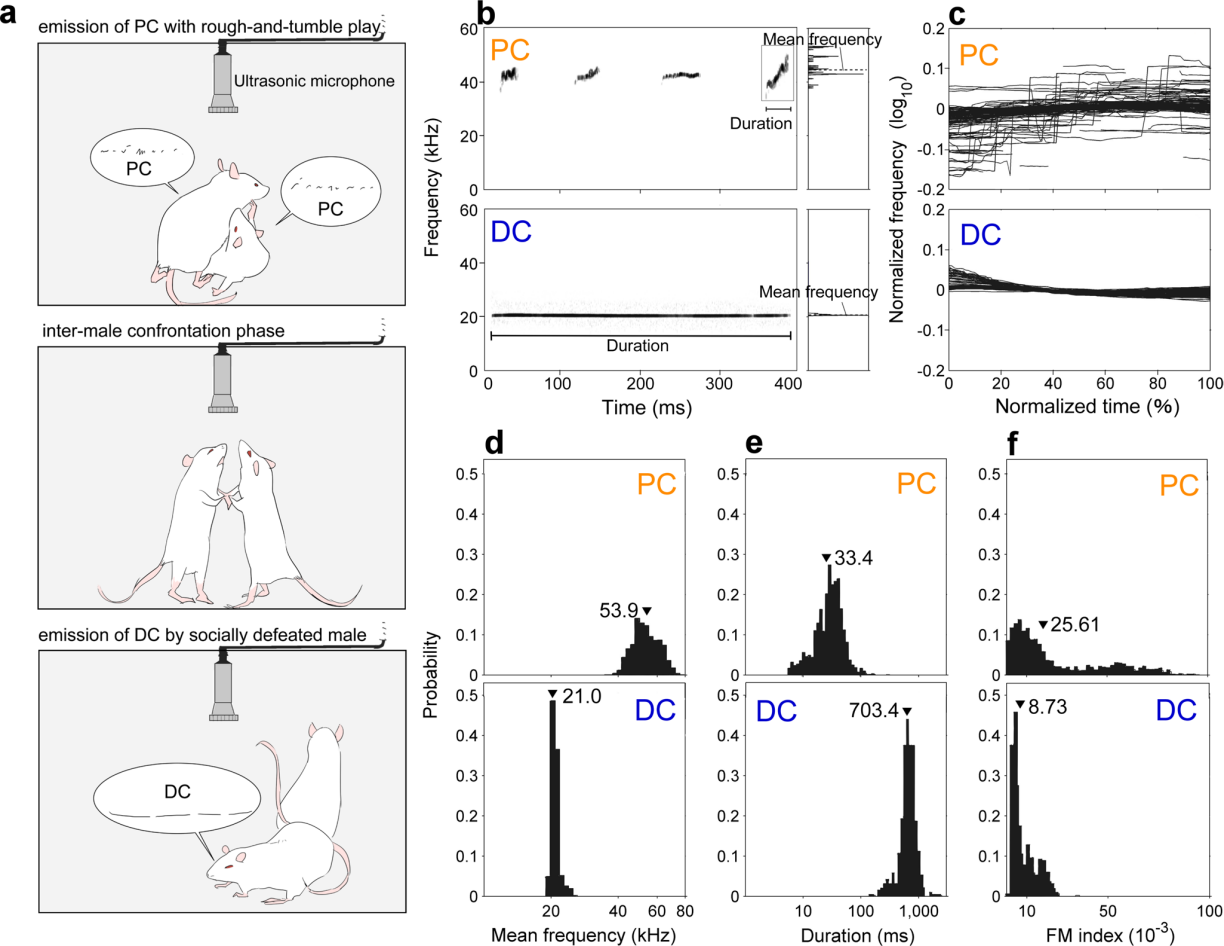
Typical examples and general properties of the three acoustical features of a pleasant call (PC) and a distress call (DC). (**a**) The process for recording PC and DC stimuli. As soon as each pair was placed in a sound-attenuated chamber, they started to emit PC with rough-and-tumble play. They continued to the inter-male confrontation phase, and then the socially defeated male emitted DC. Their vocalizations were recorded via an ultrasonic microphone placed in the chamber. (**b**) Calculation of mean frequency and duration. Four calls of PC and one call of DC are shown. Mean frequency is provided by the concentration of spectral power. Duration is the length of one call from the start to finish. (**c**) Calculation of frequency modulation (FM) index. It is defined by the coefficient of variation of frequencies. Some examples of PC and DC are shown. The x-axis represents relative lengths from 0 to 100%. The y-axis represents the normalized logarithmic frequency (the mean was uniformed to zero). These graphs show that PC have more frequency variation (i.e., have a larger coefficient of variation) than DC. (**d**-**f**) The distribution of mean frequency, duration and FM index from recorded PC (upper panels) and DC (lower panels). Arrowheads indicate the mean values. All y-axes represent the probabilities. The x-axis represents kilohertz (logarithmic) for mean frequency, milliseconds (logarithmic) for duration, and FM index was expressed in arbitrary units (because FM index is the coefficient of variation of frequency in a single call).

**Figure 2:**
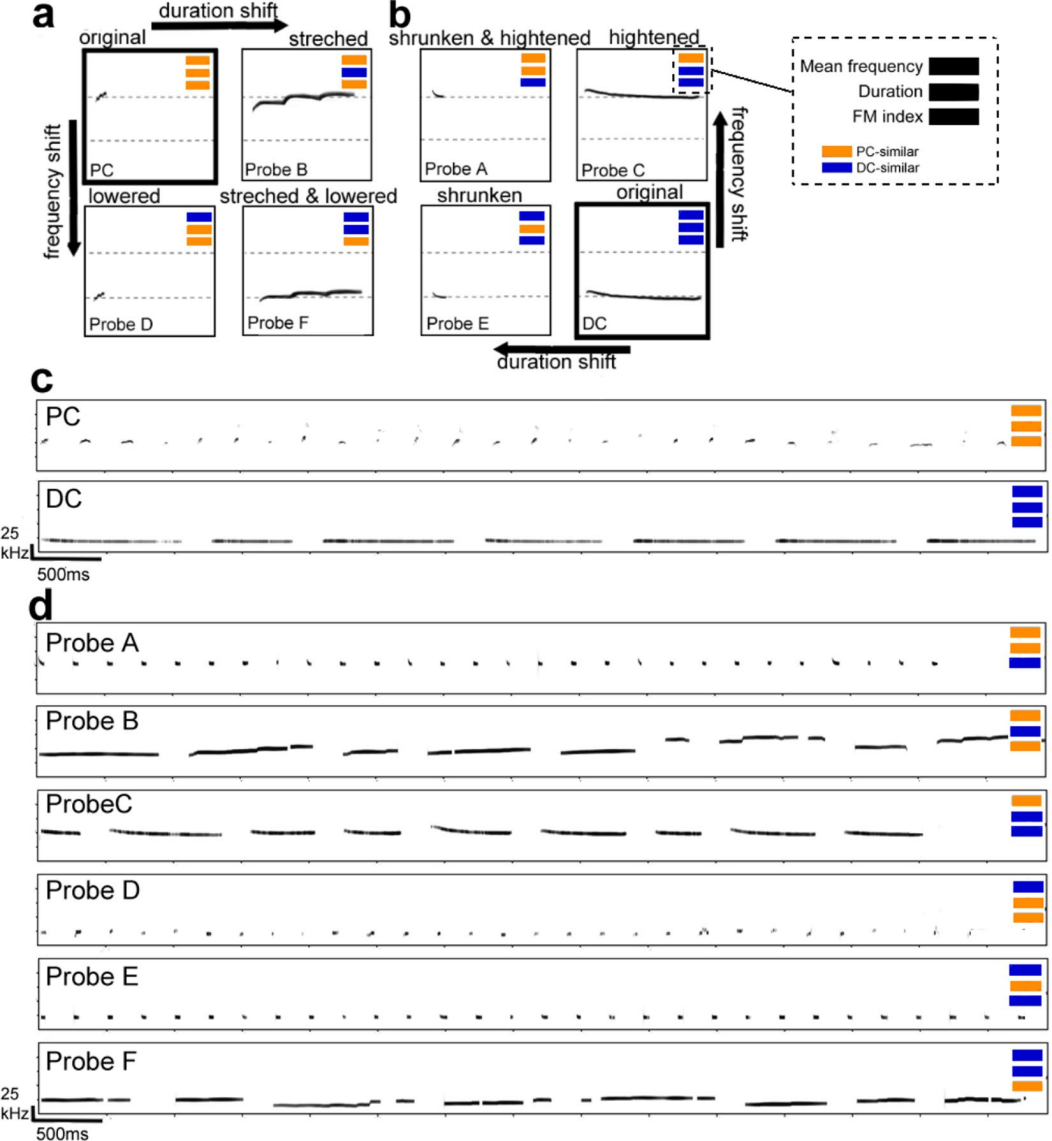
The method used to synthesize probe stimuli and example spectrograms. (**a**,**b**) The method of synthesizing probe stimuli from USVs: PC or DC. Probe stimuli are synthesized by shifting either mean frequency or duration, or both. Probe stimuli B, D and F are made from PC. Probe stimuli A, C and E are made from DC. For each stimulus, PC-similar parameters are represented as orange bands and DC-similar parameters are represented as blue bands. (**c**) Examples spectrogram of stimuli used in the training phase. For each spectrogram, the x-axis represents time, and the y-axis represents frequency. One sample of a PC stimulus, which was composed of twenty-four calls, and one sample of a DC stimulus, which was composed of seven calls. (**d**) Examples spectrograms of probe stimuli. There were six categories of probe stimuli. For example, Probe A has PC-similar mean frequency (around 50 kHz) and duration (short), and DC-similar frequency modulation (stable). See Table 1 for acoustical properties of each probe stimulus.

In order to demonstrate the relative contributions of acoustical features for auditory discrimination of ultrasonic calls in rats, we tested discriminative choices on synthesized calls whose acoustical features were systematically manipulated. We focused on three features (see Fig. 1b,c): mean frequency, duration, and an index of FM patterns (FM index). First, we analyzed these features between PC and DC. One or two of these features were swapped between PC and DC to test the impact of that feature for discrimination (probe stimuli: see Fig. 2a,b). Then, we trained rats to perform a two-alternative forced-choice test, pressing one lever when they hear PC, and the other when they heard DC. After training, animals underwent discrimination testing in which they performed the lever press for six types of probe stimuli. From this test performance, we assessed the relative contribution of acoustical features for emotional USV recognition.

### Acoustical differences between pleasant and distress calls

We first analyzed 1441 PC and 465 DC, to confirm that recorded calls had the same characteristics as those in past studies. The mean frequency was 53.9 ± 7.4 kHz (Fig. 1d) and the duration was 33.4 ± 19.6 ms (Fig. 1e) for PC. The mean frequency was 21.0 ± 0.9 kHz (Fig. 1d) and the duration was 703.4 ± 236.7 ms (Fig. 1e) for DC. These numbers were consistent with previously reported values^13,15,17^. The mean and the standard deviation of the FM index (Fig. 1f) were significantly larger for PC than for DC (PC: 25.61 ± 25.78 × 10^−3^; DC: 8.73 ± 6.04 × 10^−3^; *p* < 0.001, Welch’s t-test).

### Contribution of acoustical features for discrimination

Discrimination tests were conducted to assess the contribution of candidate acoustical features using six probe stimuli (Probes A-F) in which the acoustical features were systematically swapped between PC and DC (Fig. 2, Table 1; see detail in Methods). For example, Probe A was synthesized from recorded DC samples by both shortening the duration and increasing the frequency to have the same range of duration and mean frequency as PC while preserving the frequency modulation pattern of DC (this combination is indicated by orange/blue colored bands in Fig. 2d). Similarly, Probe F was made from PC samples but modified to have the same duration and mean frequency ranges as DC. Acoustical measurements on final products showed that these probe stimuli were properly synthesized as we intended (Table 1).

**Table 1:** The parameters of stimuli.

	Mean frequency		Duration		FM index	
	similar	M ± SD (kHz)	similar	M ± SD (ms)	similar	M ± SD (10^−3^)
PC	—	53.9 ± 7.4	—	33.4 ± 19.6	—	25.61 ± 25.78
DC	—	21.0 ± 0.9	—	703.4 ± 236.7	—	8.73 ± 6.04
Probe A	PC	53.9 ± 4.0	PC	36.6 ± 16.2	DC	7.22 ± 5.98
Probe B	PC	56.2 ± 8.1	DC	622.7 ± 287.1	PC	22.55 ± 20.95
Probe C	PC	54.5 ± 6.2	DC	683.0 ± 194.7	DC	12.34 ± 4.69
Probe D	DC	19.2 ± 2.8	PC	41.7 ± 22.1	PC	24.20 ± 24.45
Probe E	DC	20.3 ± 0.2	PC	35.9 ± 19.2	DC	11.07 ± 6.89
Probe F	DC	21.5 ± 2.2	DC	771.5 ± 309.0	PC	26.85 ± 22.25

The mean ± standard deviation of mean frequency, duration and FM index in all stimulus categories: PC and DC stimuli, and six probe stimuli. Each probe stimulus had either PC-similar or DC-similar parameters in three acoustical features.

**Figure 3:**
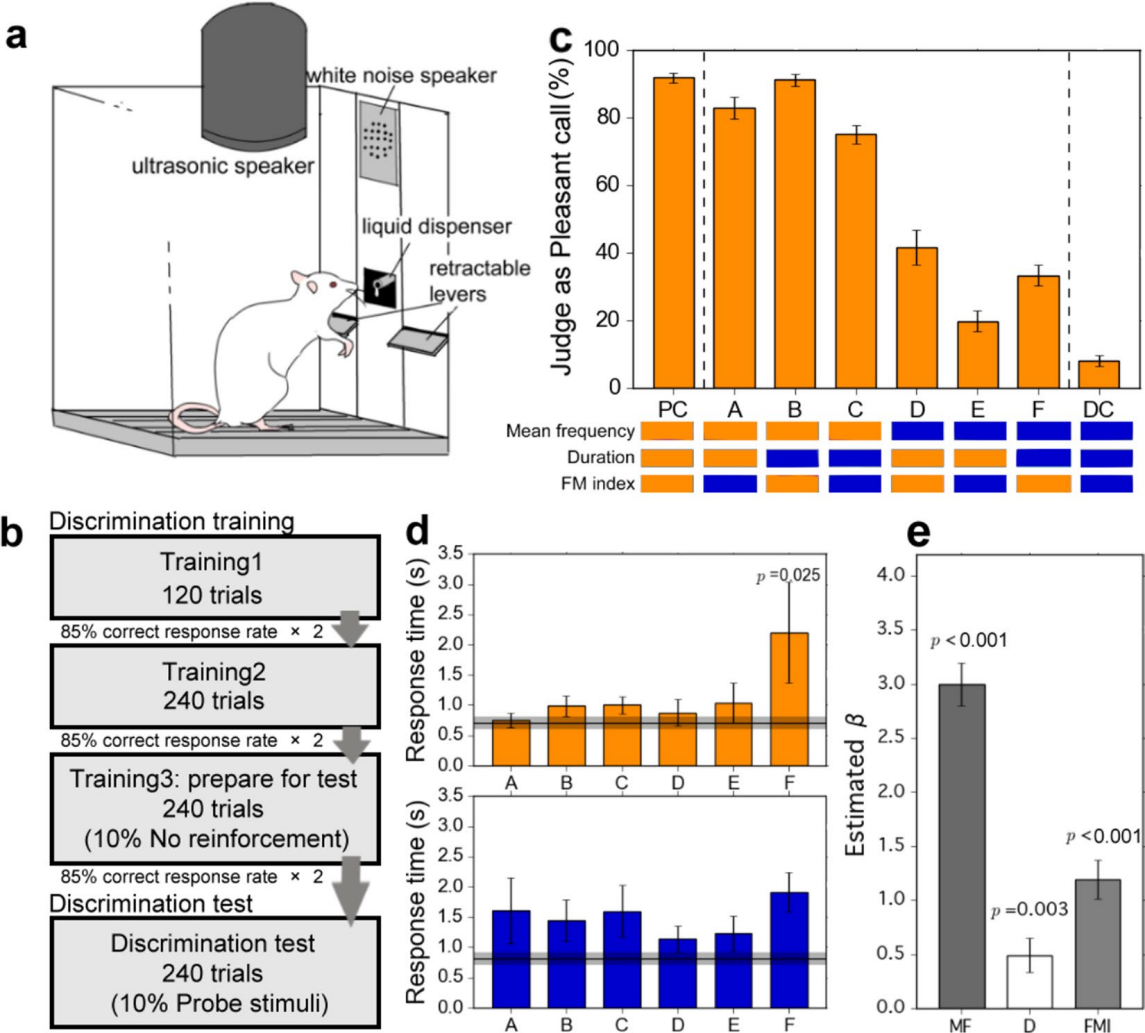
Behavioral procedures and data in the discrimination tests. (**a**) The operant chamber used in this study. The chamber was equipped with two retractable levers. The liquid dispenser, which dispensed a sucrose solution, and the white noise speaker were located between the two levers. The ultrasound speaker was fixed above the box. (**b**) A flow chart demonstrating the three trainings and discrimination test. Discrimination training occurred in three stages. In the third training, some trials in which PC and DC stimuli were not reinforced were added in order to prepare them for the probe stimuli in the discrimination tests. Animals that showed a correct response rate of more than 85% two days in a row continued to the next stage. (**c**) The rate of classification as PC for each stimulus. Bars indicate the rate of judging the probe stimulus as PC. Error bars represent the standard error. For each stimulus, PC-similar parameters are represented as orange bands and DC-similar parameters are represented as blue bands. On the presentation of PC or DC, the correct response rate surpassed 90%, but did not reach 100%. Rats tended to class probe stimuli A, B, and C as PC. On the other hand, they tended to class probe stimuli D, E, and F as DC. This indicates that mean frequency was the most salient cue for classification. In addition, Probe B was most often judged as PC, while Probe E was most often judged as DC. This suggests that FM index also had an impact on their classification. The GLMM logistic regression supports these results. (**d**) The average response time for each stimulus. All error bars represent the standard error. The upper panel shows the response time when stimuli were judged as PC. Orange bars indicate the time to press the PC lever for each probe stimulus. The horizontal line and shadow range represent the average response time and standard error for PC stimuli (correct response). Compared to other correct responses, Probe F shows a significant difference (*p* = 0.025; Dunnett’s multiple comparison). The lower panel shows the response time when stimuli were judged as DC. Blue bars indicate the time to press the DC lever for each probe stimulus. The horizontal line and shadow range represent the average response time and standard error for DC stimuli (correct response). There was no significant difference between correct responses and any probe stimuli. (**e**) The estimated coefficients by GLMM logistic regression. The PC/DC-similar mean frequency (MF) around 50 kHz/22 kHz was the dominant predictor for the categorization of PC/DC (*p* < 0.001). The PC/DC-similar variable/stable FM patterns (FM index: FMI) and short/long duration (D) also promoted the correct categorization of calls (FMI: *p* < 0.001; D: *p* = 0.003), but to a lesser extent.

After training sessions for pressing lever to discriminate between PC and DC stimuli (Fig. 3a,b), rats showed a correct response rate of above 90%, which exceeds the training criterion of an 85% correct response rate, when discriminating between PC and DC. When probe stimuli were presented, animals discriminated all stimuli as either the PC or DC by pressing either lever. The response rate for the probe stimuli showed a systematic change according to the acoustical features of the stimuli: animals judged them as PC at a rate of 82.8%, 90.6%, 72.4%, 42.2%, 19.8%, and 33.3% for Probe A-F, respectively (Fig. 3c). The rates judged as DC were simply the difference between 100% and the rate judged as PC, as PC and DC lever presses equaled 100%. Even if animals did not perceive probe stimuli as only PC or DC, our results suggest that they do respond to probe stimuli and discriminate them either PC or DC, just not exclusively.

A generalized linear mixed model (GLMM) analyzed the discrimination test data to determine acoustical factors for judging probe stimuli as either PC or DC. Using Akaike Information Criteria (AIC) and Bayesian information Criteria (BIC; see Table 2), we selected the model which included all single variables and in which there was no interaction as the best. This model showed that the having a PC/DC-similar mean frequency around 50 kHz/22 kHz was the dominant predictor for the categorization of calls as PC/DC (*β* ± SE = 3.00 ± 0.20, *z* = 15.38, *p* < 0.001; Fig. 3e). The PC/DC variable/stable FM patterns also promoted the correct categorization of calls, but to a lesser extent (*β* ± SE = 1.19 ± 0.18, *z* = 6.51, *p* < 0.001; Fig. 3e). The effect of duration could not be ignored. Having a PC/DC-similar, short/long duration facilitated the categorization as well (*β* ± SE = 0.49 ± 0.16, *z* = 3.0, *p* = 0.003; Fig. 3e).

**Table 2:** The comparison among the top five models.

	(Intercept)	MF	D	FMI	MF × D	MF × FMI	D × FMI	AIC	BIC
model 1	−1.95 (*p* < 0.001)	3.00 (*p* < 0.001)	0.49 (*p* = 0.003)	1.19 (*p* < 0.001)	—	—	—	1221.8	1247.0
model 2	−2.04 (*p* < 0.001)	3.03 (*p* < 0.001)	0.61 (*p* = 0.009)	1.33 (*p* < 0.001)	—	—	−0.23 (*p* = 0.440)	1223.2	1253.5
model 3	−1.90 (*p* < 0.001)	2.93 (*p* < 0.001)	0.42 (*p* = 0.041)	1.19 (*p* < 0.001)	0.16 (*p* = 0.605)	—	—	1223.5	1253.8
model 4	−1.91 (*p* < 0.001)	3.00 (*p* < 0.001)	0.48 (*p* = 0.003)	1.15 (*p* < 0.001)	—	0.11 (*p* = 0.747)	—	1223.7	1254.0
model 5	−2.04 (*p* < 0.001)	3.03 (*p* < 0.001)	0.62 (*p* = 0.014)	1.34 (*p* < 0.001)	—	−0.02 (*p* = 0.484)	−0.23 (*p* = 0.441)	1225.2	1260.5

MF, D and FMI indicate mean frequency, duration and FM index, respectively. Table shows intercept and coefficients of regressors, and their p-values. Comparing AIC and BIC values, the best fit model included all single regressors (MF, D, FMI) and no interaction.

We compared the average response time between PC or DC and each probe stimulus (Fig. 3d). When rats showed the correct response on the presentation of PC or DC, they responded very rapidly (PC: 0.71 ± 0.35 s; DC: 0.81 ± 0.39 s). When being judged as PC, the response time to Probe F was significantly longer than other probes judged as PC (2.20 ± 2.90 s; *p* = 0.025, Dunnett’s multiple comparison). Aside from this condition, there was no significant difference in response times. We expect that there is some ambiguity when Probe F is judged as PC.

## Discussion

Adult rats have two categories of emotional vocalizations: pleasant calls (PC) and distress calls (DC). These calls have acoustical differences in three features (mean frequency, duration and FM pattern). With the aim to learn the impact of acoustical features for auditory perception, we used psychophysical methods to systematically study rat communication calls for the first time. As a result, the strongest factor was mean frequency, followed by FM pattern (the frequency variation within calls) and duration (the length of the call from onset to offset). There was no significant interaction among the contribution of acoustical features. This result indicated that rats used all features independently for discrimination, with an emphasis on mean frequency.

The reason that mean frequency had the strongest impact on perceptual discrimination can be explained by the animal’s auditory sensitivity. Because the physical structure (specifically the size) of middle and inner ear determines the resonant frequencies, high frequency calls above 20 kHz are easily perceived by rodents’ smaller auditory organs and help them avoid being detected by larger predators^18^. Thus, they possibly acquire strong auditory detection of frequencies above 20 kHz^19^, which is the same frequency range used for communication through emotional calls. Thus, it stands to reason that perception of these calls largely depends on mean frequency.

The FM patterns and duration also had a significant impact on perception of calls, though it was much smaller than that of the mean frequency. A possible reason is that differences in frequency modulation and duration between PC and DC are generated as a byproduct of intrinsic respiratory, laryngeal, and vocal tract movement patterns used for vocalizing in different frequency bands^20^. Thus, listeners can learn the difference in FM patterns and duration, which are related to the frequency bands of the two call types. In addition, FM can enhance detectability in the sensory system by reducing the habituation effect. Natural DC has been shown to activate several brain regions such as the periaqueductal gray, lateral and basolateral amygdala, and perirhinal cortex^21,22^, whereas 20 kHz sine-wave tones or artificial calls (the same bout-structure but with straight upward frequency-modulation) were less effective in activating these regions^22^. These observations also support the idea that FM patterns in rat calls can be a cue for vocal sound discrimination.

Our study trained animals to associate PC and DC with respective response levers through reward, although it should be noted that DC has a natural tendency to become a cue for fear conditioning rather than reward conditioning^23,24^. DC could have preparedness for fear-related events or behaviors, i.e., the association between DC and fear is rapidly made^25^. However, we conclude that preparedness did not influence the results of our study because during discrimination training, animals exceeded the 85% correct criterion for detection of both DC and PC. Even in discrimination testing, animals showed equally rapid responses to both PC and DC (PC: 0.71 ± 0.35 s; DC: 0.81 ± 0.39 s) when they showed correct responses (Table 3).

**Table 3:** Response rate and response time for each stimulus.

	Judged as PC		Judged as DC	
	rate (%)	time (s)	rate (%)	time (s)
PC	91.8	0.71 ± 0.35	8.2	1.65 ± 1.01
DC	8.0	1.18 ± 0.56	92.0	0.81 ± 0.39
Probe A	82.8	0.75 ± 0.42	17.2	1.61 ± 1.72
Probe B	90.6	0.99 ± 0.62	9.4	1.45 ± 1.14
Probe C	72.4	1.00 ± 0.50	27.6	1.60 ± 1.49
Probe D	42.2	0.88 ± 0.75	57.8	1.14 ± 0.77
Probe E	19.8	1.04 ± 1.16	80.2	1.24 ± 0.98
Probe F	33.3	2.20 ± 2.90	66.7	1.91 ± 1.13

The table shows the response rates to all stimuli in discrimination tests: In any one stimulus category, the rate at which the stimulus is judged as PC or DC sums to equal 100%. Mean ± standard deviation of response time is also shown.

In this study, we defined PC as calls during rough-and-tumble play without subdividing into potential categories, even though some studies have reported that PC could be divided into several subtypes, e.g., constant frequency (or flat) calls and frequency modulated calls^16^. Although others have suggested the possibility that these subtypes were selectively produced in different contexts^26^, recorded PC in our study were indistinguishable in their acoustic characteristics, based on vocalization context.

In conclusion, our test using systematically manipulated parameters of calls has found that rat auditory perception relied mostly on the mean sound frequency. In addition, duration and FM pattern also contributed to discrimination between PC and DC, which indicated that rats perceived their calls based on these features as well. This suggests that rats use mean frequency as the main source of information and use FM patterns and duration as ancillary information. The results of the present study have laid the foundation for future studies on the mechanism of perceiving emotional signals by measuring physiological responses to the probe stimuli used here.

## Methods

### Animals

Eighteen male Sprague–Dawley rats, obtained from a breeding company (Japan SLC, Inc., *Shizuoka, Japan*), were used in the study. Among them, six were used for USV recording sessions. Twelve underwent discrimination training and tests. Rats were 9–12 weeks old at the beginning of experiments, and kept in breeding cages in the same room under a 12/12-h light/dark cycle (lights on at 8:00 a.m.). The temperature was kept from 20 to 22 °C. They were fed at libitum for 7 days, their body weight was measured during this time, and it was between 312 g and 423 g. During the training and test periods, access to food was restricted (14–22 g of food per day; Lab Diet, PMI Nutrition International, *Missouri, the USA*) to maintain 85 ± 2.5% of the free-feeding weight for each rat. This was calculated from a weekly body weight measurement by fitting the standard growth curve^27^. All experimental protocols in the present study were approved by the animal experimental committee at the University of Tokyo (permission #27–8) and were performed in accordance with relevant guidelines and regulations.

### Vocalization recordings

We recorded ultrasonic vocalizations from three pairs of rats, which did not participate in the discrimination training or tests. As soon as each pair was placed in a sound-attenuated chamber (interior: 55 cm (L) × 30 cm (W) × 35 cm (H)), they started to emit PC with rough-and-tumble play^2^ (Fig. 1a). Two out of three pairs continued the inter-male confrontation phase, and then the socially defeated male emitted DC^5^ (Fig. 1a). Their vocalizations were recorded via an ultrasonic microphone (UltraSoundGate, Avisoft, *Glienicke, Germany*) placed in the chamber, and digitized by recording software (Recorder-USGH, Avisoft) at a 250-kHz sampling with 16-bit resolution. These vocalizations were recorded within 30 minutes.

### Acoustical characterization of vocalizations

We assessed acoustical differences between PC and DC on three characteristic acoustical features: mean frequency, duration, and frequency-modulation index (FM index). First, we segmented calls from continuously recorded sound data, and extracted the peak frequency which had the highest amplitude in the spectrum for each time frame within the segmented call (peak frequency trace) using MATLAB-based software ‘USVSEG’^28^. Then, the mean frequency was calculated as an average of the peak frequencies of different time frames within each call (Fig. 1b). The duration was the length of time between the onset and offset of the call. The FM index was defined to reflect the degree frequency modulation in the call irrespective of its frequency range or duration. For this purpose, we normalized the frequency and time of the peak frequency trace for each call to obtain a residual FM pattern after removing the influence of frequency range and duration. Frequency values of the peak frequency trace were divided by the mean frequency and converted into logarithmic scale, and time points were divided by the duration to be ranged from 0 (onset) to 1 (offset). Then, the standard deviation of the normalized frequency values was defined as the FM index, which increases its value when the original peak frequency trace has rapid and wider fluctuations in frequency.

### Sound stimuli

All stimuli used in this study were recorded or synthesized ultrasonic vocalization sounds containing multiple calls, with an overall duration of around 7 seconds (Fig. 2). We presented the recorded PC or DC when training animals to discriminate between them, and then presented other synthesized calls during the discrimination tests. Thirty PC stimuli (27.70 ± 2.05calls, 7.14 ± 0.24 s in duration for each stimulus) and thirty DC stimuli (7.37 ± 1.18 calls, 7.09 ± 0.26 s) were used for training (Fig. 2c) after noise reduction by sound editing software (SASLAB pro, Avisoft).

To assess the impact of acoustical features in the discrimination tests, we synthesized probe stimuli from the recorded vocalization sounds by swapping either mean frequency or duration, or both, between PC and DC (Fig. 2a,b). There were six categories of probe stimuli (Fig. 2d). Table 1 shows the mean and standard deviation of the mean frequency, duration, and FM index in each stimulus category. To verify synthesized stimuli do not include any artifacts, we compared animal responses to original PC/DC stimuli and synthesized PC/DC stimuli (100% shifting of mean frequency and duration from original stimuli) after discrimination testing (see Supplementary information: Additional results).

All stimuli were played back from an audio amplifier (Integrated-amplifier A-10, Pioneer, *Kawasaki, Japan*) and an ultrasound speaker (PT-R4, Pioneer). Note that all stimuli were presented at random levels from 55 to 75 decibels sound pressure level (dB SPL), although recorded DC had larger sound levels than PC. This was because the distance between callers and listeners when emitting DC was longer than for PC. It is unlikely that sound levels influenced discrimination performance because animals were trained to respond to the auditory stimuli and not the level, and the sound levels were randomized to normalize any differences between the stimuli.

### Discrimination training

To train for auditory discrimination between PC and DC, each rat was placed in an operant conditioning chamber (interior: 29.53 cm (L) × 23.5 cm (W) × 27.31 cm (H), ENV-007-CT, Med Associates, *Vermont, the USA*). The chamber was sound-attenuated and equipped with a light, a white noise speaker, a liquid dispenser, a grid floor and two retractable levers. The liquid dispenser, which dispensed a sucrose solution, was located between the two levers. The ultrasound speaker was fixed above the box (Fig. 3a). Prior to conducting the training phase, rats were placed inside the box two or three times to habituate them to the chamber. During this time, they also learned to press the left and right levers to gain access to the sucrose solution.

Discrimination training occurred in three stages (Fig. 3b). During all stages, rats were trained to press one lever when a PC stimulus was presented (PC lever) and the other lever when a DC stimulus was presented (DC lever). All ultrasonic sound stimuli were randomly presented through the operant chamber controlling system (DIG-700F, DIG-716, DIG-726TTL-G, Med Associates; Power 1401, CED, *Cambridge, the UK*). After the presentation of a stimulus, two levers were presented and rats were forced to choose one lever. They received a reward (20% sucrose solution dispensed for 2 s) when they pressed the correct lever. When they pressed the incorrect lever, they received an aversive 90 dB SPL white noise for 30 s and the same trial was continued in order to correct the false response. Rats could not advance to the next trial until they pressed the correct lever. As a consequence of this discriminative training, they learned to press the correct lever when they recognized PC or DC, respectively.

In the initial training, ten of thirty PC stimuli and ten of thirty DC stimuli were used. Within a 60-minute period, rats performed 120 trials. Trials were separated by a 10 s inter-trial interval (ITI). Training sessions occurred once per day. During the early days of training, nearly all rats were unable to finish the 120 trials within the 60-minute time period. In these cases, training terminated at the 60-minute time limit. However, within one month, rats were able to shape their discriminative responses to the two stimuli (16.5 ± 3.9 days). Rats that showed a correct response rate of more than 85% two days in a row continued on to the second stage of training.

In the second stage of training, twenty of thirty PC stimuli and twenty of thirty DC stimuli were used. There were 240 trials within a 120-minute period. Rats that showed a correct response rate of more than 85% two days in a row continued on to a third stage of training. All rats achieved the criteria within 4 days.

In the third stage of training, all thirty PC and DC stimuli were used. There were 240 trials within a 120-minute period. In 10% of trials, no reward or punishment was given regardless of which lever rats pressed. These trials were added in order to prepare them for the probe stimuli in the discrimination tests. Thus, 108 trials of PC stimuli, 108 trials of DC stimuli, and 24 non-reinforced trials were prepared in this stage. If a rat showed a correct response rate of more than 85% two days in a row in the third training stage, we continued on to the discrimination tests. Although the probe stimuli were novel for the rats, none of them ever failed in these sessions. Thus, rats were not only able to discriminate between PC and DC, but they were also able to generalize novel stimuli to the same categories of USVs.

### Discrimination tests

To assess the contribution of acoustical features for discrimination, discrimination tests were conducted. All thirty PC and DC stimuli were used in discrimination tests. In addition, probe stimuli were presented in 10% of all trials (24 trials: 4 trials for each probe stimulus category). There were 240 trials within a 120-minute period. As probe stimuli consisted of six categories, stimuli in one category were presented four times in one session. During the session, we recorded the proportion of pressing the PC or DC levers in each trial. After test sessions, rats continued to perform the third training for one week and were given a second discrimination test a week later in order to avoid habituation to the probe stimuli. These tests were carried out four times for one individual in total. The behavioral responses and times were recorded by the chamber’s control system (DIG-700F, DIG-716, DIG-726TTL-G, Med Associates).

### Statistical tests

All data were statistically analyzed using R version 3.5.1 (R Foundation for Statistical Computing, *Vienna, Austria*) with the lme4 package^29^ and multcomp package^30^. For assessing differences in the FM index (an acoustical feature reflecting frequency-modulation rate) between recorded PC and DC vocalizations, we used Welch’s t-tests with a significance level set at 0.05. For statistical assessment of the discrimination test data obtained from 12 animals, we used the generalized linear mixed model (GLMM) approach for the logistic regression of binary responses. As described above, we presented probe stimuli in which we systematically swapped the acoustical features between PC and DC to assess perceptual impacts of these features on PC/DC discrimination. Thus, each binary response (PC- or DC-lever press) could be predicted by binary independent variables representing whether the acoustical features of the probe originated from PC or DC (as shown by the orange-blue colored bands in Figs 2 and 3). For this reason, the three acoustic parameters: mean frequency, duration and FM index were treated as dummy variables that had two categorical values: PC-similar or DC-similar. The binary response of animals to press one of the two levers was set as the responsive variable. We treated the dummy variables for the three acoustical features as fixed factors, and the individual difference as a random factor. Using the Bayesian information criteria, the best fit model was searched for among regression models consisting of these variables and the interaction terms. We additionally used the Akaike information criteria, to check the consistency of the model selection results. Each coefficient was estimated by the method of Laplace approximation^29^. In addition, response times to press the lever for the probe stimuli were compared with that for recorded PC or DC stimuli in order to confirm that animals treated the probe stimuli in a similar way as the natural PC/DC stimuli. The statistical significance of the response time difference was tested by Dunnett’s multiple comparison with a significance level set at 0.05.

## Supplementary information

supplementary infomation

## Data Availability

The datasets generated during and/or analyzed during the current study are available in the *figshare* repository, 10.6084/m9.figshare.8241290.
