# Supplementary material for: Acoustical cues for perception of emotional vocalizations in rats
Source: Sci Rep. 2019 Jul 22;9:10539. doi: 10.1038/s41598-019-46907-0 (PMC6646302; doi:10.1038/s41598-019-46907-0)
Supplement: Supplementary file 1 — supplementary infomation [file 41598_2019_46907_MOESM1_ESM.docx]

# Supplementary information

Title

Acoustical cues for perception of emotional vocalizations in rats

Author lists

Yumi Saito^1^

Ryosuke O. Tachibana^1^

Kazuo Okanoya^1^

1 Department of Life Sciences, Graduate School of Arts and Sciences, The University of Tokyo, 3-8-1 Komaba, Meguro-ku, Tokyo, Japan

Correspondence author:

Kazuo Okanoya

**Additional methods**

## Spectral peak detection

To assess acoustical features of rats’ vocalizations, we extracted peak frequency traces of each vocalized sound after performing noise reduction and segmentation on the recorded sound dataset. We detected each syllable from continuously recorded sound data by in-house software ('usvseg') implemented as MATLAB scripts^1^. Briefly, the original waveform was converted into a smoothed spectrogram by the multitaper method^2^ with a 4-ms window (1024 samples) in 0.5-ms timesteps (125 samples); background noise (chamber fan, scratching sounds, etc.) was reduced by cepstral filtering (or liftering) and median subtraction. Then, amplitude and frequency of the spectral peak in every timestep were calculated as the maximum amplitude of the spectrum and a weighted mean of 21 spectral points around a frequency corresponded to the maximum amplitude.

## Artifact checking for auditory stimuli

We also conducted tests to compare the responses toward original PC/DC stimuli (used for training and discrimination tests) and an additional set of synthesized PC/DC stimuli (100% shifting of mean frequency and duration from originals) in order to ensure that synthesized stimuli (i.e., probe stimuli) did not have any artifacts. Synthesized PC/DC stimuli were created by the same program that was used for probe stimuli synthesis, by 100% shifting of mean frequency and duration from the original stimuli. So values of three acoustical features were entirely the same between original PC/DC and synthesized PC/DC. After all discrimination test sessions were finished, six of twelve rats were tested once. There were 240 trials within a 120-minute period, and 10% of trials were the presentation of synthesized PC/DC stimuli without rewards or punishments. Then we checked whether rats showed the correct response to synthesized PC/DC. We also compared the response time lever press by two sample t-tests.

**Additional results**

## Auditory stimuli did not have any artifacts

To ensure that synthesized stimuli did not have artifacts, we calculated the correct response rate (the rate of pressing PC or DC levers during the presentation of original or synthesized PC or DC stimuli, respectively). When synthesized PC/DC stimuli were presented, the correct response rate surpassed the 85% criterion (93.1 % for synthesized PC, 97.2 % for DC). In addition, there were no significant differences in response times for between natural and synthesized stimuli (*p* > 0.1). Thus, we conclude our method of audio synthesis did not introduce any artifacts into the stimuli.

Supplementary references

1. Tachibana, R. O., Kanno, K., Okabe, S., Kobayasi, K. I., & Okanoya, K. USVSEG: A robust segmentation of rodents' ultrasonic vocalization. BioRxiv, 572743 (2019).
2. Thomson, D. J. Spectrum estimation and harmonic analysis. *Proceedings of the IEEE.* Vol. 70, pp. 1055–1096 (1982)
